# Assessment of *human Herpes Virus-8* infection in Iranian cirrhotic patients on the waiting list for liver transplantation: A cross-sectional analysis

**DOI:** 10.1016/j.nmni.2024.101496

**Published:** 2024-10-02

**Authors:** Javad Moayedi, Ava Hashempour, Zahra Musavi, Farzaneh Ghasabi, Nastaran Khodadad, Mohamad Ali Davarpanah, Ali Hasanshahi

**Affiliations:** HIV/AIDS Research Center, Institute of Health, Shiraz University of Medical Sciences, Shiraz, Iran

**Keywords:** Antibody, Cirrhosis, HHV-8, Liver transplantation, Mimicry, Viral load

## Abstract

**Background:**

*Human Herpes Virus 8* (*HHV-8*) is involved in autoimmunity. However, its association with advanced liver disease has not been fully explained. Herein, the prevalence of *HHV-8* viremia was assessed in Iranian liver transplant candidates with a confirmed diagnosis of cirrhosis.

**Methods:**

This cross-sectional study was conducted on 230 patients with cryptogenic cirrhosis, virus-related cirrhosis, and autoimmune hepatitis, as well as 140 healthy blood donors from April 2022 to September 2023. The *HHV-8* IgG antibody concentration and viral load were evaluated *via* ELISA and RT‒PCR, respectively.

**Results:**

Anti-*HHV-8* IgG antibodies were detected in 25 cirrhotic patients (10.8 %) and four healthy individuals (2.6 %) (p = 0.022). The majority of the seropositive patients had cryptogenic cirrhosis (20.4 %), followed by autoimmune hepatitis (13.1 %) and virus-related cirrhosis (4.7 %). The seropositivity of *HHV-8* IgG antibody was significantly different among the etiologies of liver cirrhosis (p = 0.011). However, *HHV-8* genomic DNA was not detected in the sera of the patients or healthy blood donors.

**Conclusion:**

The role of *HHV-8* infection in the development of posttransplant diseases, together with the higher seroprevalence of *HHV-8* antibodies in cirrhotic patients than in healthy individuals, highlights the importance of both primary and latent infections in liver transplantation. Therefore, serological and molecular screening of *HHV-8* is highly suggested for liver transplant candidates and organ donors. The possibility of antibody-mediated epitope mimicry in cryptogenic and autoimmune groups with moderate *HHV-8* antibody positivity and negative viral loads may account for the development of advanced liver diseases.

## 1 Introduction

Liver cirrhosis represents a significant proportion of end-stage liver disorders and is considered one of the main causes of morbidity and mortality worldwide. It is characterized by the formation of regenerative nodules surrounded by extensive tissue fibrosis [1,2]. Cirrhosis invariably leads to death unless liver transplantation is performed. The etiology of liver cirrhosis varies geographically. The most common causes in Western countries include heavy alcohol consumption, chronic *hepatitis C virus* (*HCV*) infection, and nonalcoholic fatty liver disease (NAFLD), whereas the primary cause is chronic *hepatitis B virus* (*HBV*) infection in the Asia–Pacific region and Iran. Other common causes of liver cirrhosis include hemochromatosis, Wilson's disease, primary biliary cirrhosis, primary sclerosing cholangitis, and autoimmune hepatitis [[3], [4], [5], [6]]. Notably, some cases of cirrhosis are idiopathic or cryptogenic, which refers to liver cirrhosis of undetermined etiology with a lack of definitive clinical and histological criteria [7,8]. Viral hepatitis, especially *HBV* and *HCV*, has been suggested to be the main cause of virus-related cirrhosis. However, the roles of other viruses in the development and progression of liver cirrhosis have not been fully elucidated.

*HHV-8* is a large, double-stranded deoxyribonucleic acid (DNA) virus with a high level of genetic variability. Two modes of infection are supported: primary *HHV-8* infection is asymptomatic and rarely diagnosed, but the virus can infect B cells and establish a latent state in the host, which is a typical characteristic of all herpesviruses [9]. *HHV-8* is a human oncogenic virus known as Kaposi's sarcoma-associated herpesvirus (*KSHV*), highlighting its public health significance [10]. Although the exact routes of transmission are not fully understood, *HHV-8* is thought to be transmitted by direct contact with blood (transfusions) or other bodily fluids, including saliva and semen. It can also be reactivated and transmitted through transplantation [11,12]. Studies have reported that the risk of *HHV-8* infection increases in families of *KSHV* patients (with horizontal and/or vertical transmission), healthcare personnel, individuals undergoing unprotected sex, blood transfusions and dialysis and individuals in close fecal contact with infected people [[13], [14], [15]]. The risk of Kaposi's sarcoma (KS) is highest within 30 days posttransplantation. Nonetheless, the average time from transplantation to the development of KS is approximately 6.2–10.4 months [12]. In addition, primary effusion lymphoma, a large B-cell neoplasm, is universally associated with *HHV-8* infection in the context of immunodeficiency [16].

Although the association between *HHV-8* infection and advanced liver diseases such as cirrhosis has remained unclear, some studies have shown that, compared with healthy individuals, cirrhotic patients have a greater frequency of *HHV-8* infection [17,18]. Liver cirrhosis is associated with alterations in both innate and acquired immunity and leads to immunodeficiency. Immunological disturbances with impaired immune function in such patients may increase the risk of *HHV-8* infection [19]. In addition, primary infection and/or reactivation of *HHV-8* infection are sometimes associated with severe or fatal complications in immunosuppressed patients, especially after organ transplantation [20]. In this context, the current study aimed to assess the prevalence of *HHV-8* infection among Iranian cirrhotic patients waiting for liver transplantation. This study focused on patients with cryptogenic cirrhosis, virus-related cirrhosis, and autoimmune hepatitis, which are the main indications for liver transplantation in the Iranian population.

## 2 Materials and methods

### 2.1 Patient selection

A total of 230 liver transplant candidates who were admitted to the organ transplant unit at Nemazee Hospital, the largest liver transplant center in the world, affiliated with Shiraz University of Medical Sciences, Shiraz, Iran, during the study period from April 2022 to September 2023 were enrolled in this cross-sectional study. All cirrhotic patients were routinely visited by the physicians of this practice. The inclusion criteria were 1) providing a confirmed diagnosis of cirrhosis due to cryptogenic cirrhosis, virus-related cirrhosis (HBV- and HCV-infected individuals), and autoimmune hepatitis and 2) being a candidate on the waiting list for liver transplantation. The following criteria were used to diagnose liver cirrhosis: clinical criteria: some of the symptoms were fatigue, jaundice, and ascites; the majority of the patients presented with no clinical manifestations. Furthermore, liver function is also evaluated in terms of the bilirubin level, albumin level, prothrombin time, ascites and hepatic encephalopathy [21]; biochemical and imaging criteria: these criteria are based on hepatic recompensation and recommend individual therapies for the underlying etiology, such as antivirals for viral hepatitis [22]. Moreover, patients with a serum albumin concentration >36 g/L and a platelet count >120,000/μL may not need endoscopy for high-risk varices [23]. In brief, cirrhosis in all patients was assessed clinically, radiologically, biochemically, and histologically. The diagnosis was confirmed by liver biopsy of the explanted liver [24]. Some of the patients’ electronic medical records were compared with their physical medical records as a cross check. Diagnostic criteria of autoimmune hepatitis are based on clinical features, coupled with serological markers such as elevated transaminases and immunoglobulin G levels, autoantibodies such as ANA or SMA, interface hepatitis on liver biopsy and a positive response to immunosuppressive treatment. In this study, the criteria for autoimmune hepatitis diagnosis were a combination of the following factors, which are similar to those used in other centers [25,26]. First, the clinical features of autoimmune hepatitis usually include nonspecific features such as jaundice, fatigue, and abdominal pain, particularly in female patients [27]. Second, biochemical markers are including increased immunoglobulin G levels (>1 g/dL), elevated serum transaminases (>1x upper limit), and the presence of autoantibodies (e.g., ANA or SMA) [28]. Third, histological assessment that involved liver biopsy which is essential. Additionally, histological features, including interface hepatitis, plasma cell infiltration, and lymphocytic cholangitis, are also important key factors. The presence of these features varies across different scoring systems [29].

Certain exclusion criteria were applied in the present study, including patients with a history of previous transplantation; those suffering from liver cirrhosis with more than one etiologic agent; those with proven evidence of hepatocellular carcinoma; those with a history of HHV-8 infection; those with previous severe comorbid conditions unrelated to liver disease, such as kidney, heart, and lung diseases; those with malignancy; those younger than 18 years; those with incomplete demographic and clinical datauncooperative patients; and cirrhotic patients with other causes of liver disease (other groups), which are described in the following paragraph. Various etiologies are considered the main causes of liver cirrhosis, comprising HCV infection, HBV infection, acute liver failure, nonalcoholic steatohepatitis (NASH), autoimmune hepatitis, primary sclerosing cholangitis (PSC), alcoholism, primary biliary cholangitis (PBC), Budd-Chiari, and Wilson [24]. Patients with uncommon etiologies, including Caroli's disease, Crigler-Najjar, fibromatosis, alpha-1 antitrypsin deficiency, hemangioma, colorectal metastasis, and amyloidosis, were classified into the “other group.” Because of the limited number of patients included in this group, a quantitative analysis of the data could not be conducted; therefore, we did not include these patients in this study. The lack of direct studies focusing on exclusion criteria for HHV-8 infection concerning liver conditions limits our understanding of its pathogenic potential in hepatic contexts.

The control group included 140 healthy adult Iranian individuals of both genders who were seronegative for *HBV*, *HCV*, and *human immunodeficiency virus* (*HIV*) infection. Demographic and clinical data were obtained from the patients’ records and entered into a standard form. In this research, we chose the control group on the basis of the criteria of the patient group (case-by-case matching with no significant differences), and we also randomly selected the control group. The research protocol was approved by the local Ethics Committee of Shiraz University of Medical Sciences (approval nos. IR.SUMS.REC.1397.772 and IR.SUMS.MED.REC.1402.395).

Patient confidentiality is a fundamental ethical obligation in healthcare. In other words, confidentiality is a cornerstone of medical ethics, ensuring that patients feel safe in disclosing sensitive information, which is crucial for effective diagnosis and treatment [30]. Therefore, explicit statements regarding maintaining patient confidentiality are essential to foster trust and protect participants' rights across various medical disciplines. For this purpose, in this research, all the participants were required to provide written informed consent under the ethical framework provided by the Declaration of Helsinki.

### 2.2 Specimen collection

Whole blood samples were collected in nonheparinized tubes and allowed to clot at room temperature (20–25 °C). The samples were centrifuged at 3000 RPM for 5 min within 2 h of collection to prevent sample degradation, and then the serum fraction was separated, coded, and stored at −70 °C until use (for several months to years). The subsamples were aliquoted into smaller portions before freezing, and to increase the stability of the environmental conditions, we used a separate freezer. The samples were heated at room temperature or 4 °C to reduce shock, and to determine the reliability of the samples, each freeze-thaw for tracking purposes was documented.

### 2.3 Enzyme-linked immunosorbent assay

A commercially available enzyme-linked immunosorbent assay (ELISA) kit (*KSHV*/*HHV-8* IgG Antibody ELISA Kit, Advanced Biotechnologies Inc., Eldersburg, Maryland, USA, Cat. No. 15-501-000) was used to detect anti-latent *HHV-8* IgG antibodies in both the patient and control groups. The assay was performed following the manufacturer's instructions. All the kit components were stored at 2–8 °C until use. Prior to testing, 10 μL of the samples or controls were mixed with 990 μL of 1X specimen diluent to obtain a 1:100 dilution. Subsequently, 100 μL of the diluted samples or controls were added to a 96-well plate coated with solubilized KSHV/HHV-8 purified whole virus antigen. For the blank wells, 100 μL of 1X specimen diluent was used. Human plasma samples with and without an IgG antibody against KSHV/HHV-8 (HIV antibody negative) were utilized as positive and negative controls, respectively. The plate was sealed with an adhesive plate sealer and then incubated at 37 °C for 30 min. After the well contents were aspirated *via* an automated plate wash system, each well was washed three times with 300 μL of 1X ELISA wash buffer (PBS-Tween), with no delays between washes. Following the final wash, the plate was thoroughly tapped on clean absorbent paper to remove any remaining wash buffer from the wells. Goat anti-human IgG conjugated to the enzyme horseradish peroxidase (HRP conjugate) was then added to the wells (100 μL), and the plate was incubated for another 30 min at 37 °C. The plate was washed again to remove any unbound conjugate, as previously described. Next, 100 μL of the HRP substrate tetramethylbenzidine (TMB) was added to each well, and the plate was incubated in the dark at room temperature (22 °C) for 30 min. The enzyme‒substrate reaction was stopped by adding 100 μL of stop solution (1 N sulfuric acid). Finally, the plate reader (Epoch Microplate Spectrophotometer, Biotek, Winooski, USA) was blanked on the reagent blank wells, and the optical density (OD) was measured spectrophotometrically at 450 nm and 650 nm. The mean OD readings of three negative control wells were multiplied by 3.0 to obtain the cutoff value. The OD ratios were calculated by dividing the reading of each sample well by the cutoff value. The samples were considered positive or negative if the OD ratio was ≥1.00 or ≤0.75, respectively. Research specialists not only checked all the steps of the kit and ELISA procedures before and during the test but also routinely calibrated them with an ELISA reader; therefore, the ELISA was conducted accurately.

### 2.4 DNA extraction and real-time polymerase chain reaction

The Invisorb® Spin Virus DNA Mini Kit (Stratec Biomedical, Germany) utilizes Invisorb® technology to extract and purify high-quality viral DNA from cerebrospinal fluid, plasma, serum, etc., for in vitro diagnostic applications in a spin‒filter configuration. Fresh or frozen plasma or serum obtained from blood combined with anticoagulants such as EDTA or citrate, excluding heparin, can be used. First, 200 μl of the sample was placed into a 1.5 ml tube, followed by the addition of 200 μl of lysis buffer, which was then mixed by pipetting five times. The microtube was incubated for 5 min at room temperature with continuous shaking. After that, 20 μl of proteinase K and 20 μl of Carrier RNA were added, followed by vortexing the microtube for 10 s and incubating it for 15 min at 56 °C with continuous shaking on a thermomixer. Finally, 200 μl of binding buffer HL was added and mixed by pipetting up and down five times. The detection of *HHV-8* genomic DNA was subsequently performed *via* quantitative real-time polymerase chain reaction (PCR) according to the manufacturer's protocol (genesig standard kit, primer design, UK). This kit contains primers with 100 % homology with more than 95 % of the reference sequences for *HHV-8*, including U75698, U93872, and AF148805. A reaction mixture containing 10 μL of 2X qPCR master mix, 1 μL of HHV8 primer/probe mixture, and 5 μL of DNA template was prepared. The total volume was adjusted to 20 μL with RNase/DNase-free water. Standard precautions were followed throughout the real-time PCR assay to avoid contamination. To reduce the probability of contamination, sample preparation was performed in a different area from DNA extraction to amplification preparation. Additionally, the processes of sample extraction and analysis of amplified products were conducted in distinctly separate rooms. All procedures were performed in still-air cabinets that had to be cleaned with 5 % bleach and exposed to UV light for approximately 30 min before work began. The necessary reagents for the extraction procedure were organized within a designated room devoid of any samples. Subsequent to their opening, they were aliquoted into an appropriate volume and preserved frozen to prevent any potential contamination. On the other hand, the proper pipetting technique is one of the critical conditions that should be followed to reduce contamination to avoid the spattering of liquid and the formation of aerosols. Finally, since some samples were removed from the centrifuge tubes, it was important to spin them first and gently open the cap to control any splashing of the fluid, and the tubes were also sealed as soon as they were used. Two negative controls were utilized to check for contamination during the process of DNA extraction. Furthermore, several reactions were prepared in a single large mixture known as a master mixture. This aids in the minimization of reagent transfer, hence minimizing the chances of contaminating the reactions. Before the master mixture was prepared, the gloves were changed before using the cabinet. The negative controls included all the components incorporated in the PCR mixture except for the template, which was replaced with water. The inclusion of a negative control in PCR amplification reactions enables the detection of any contaminations present in the sample, reagent, or laboratory surroundings. The real-time PCR assay was initiated on a 7500 PRISM machine (Applied Biosystems) under the following conditions: 95 °C for 2 min, followed by 50 cycles at 95 °C for 10 s and 60 °C for 60 s. The amplification of the target sequence was detected through the FAM channel.

### 2.5 Statistical analysis

All the statistical analyses were carried out using the Statistical Package for the Social Sciences, version 22 (SPSS Inc., Chicago, USA). The normality of the data was assessed *via* the Kolmogorov–Smirnov test. Continuous variables are expressed as the means ± standard deviations (SDs) and were compared across the study groups *via* the independent samples *t*-test. Categorical variables are expressed as counts and percentages and were compared *via* Pearson's chi-square test (χ2) or Fisher's exact test, where appropriate. All reported probabilities (p values) were two-sided, and a p value less than 0.05 was considered to indicate statistical significance.

## 3 Results

The patient group consisted of 197 males (85.7 %) and 33 females (14.3 %) aged 22–76 years (mean age 57.3 ± 11.6 years). The control group included 121 males (86.4 %) and 19 females (13.6 %), with a mean age of 55.1 ± 10.3 years (range: 20–73 years). Therefore, there was no significant difference between the patient and control groups in terms of age or sex distribution (p > 0.05). The demographic characteristics of the study population are presented in Table 1.

**Table 1:** The demographic characteristics of the study population.

Variables		Liver transplant candidates				Healthy blood donors (n = 140)
		Cryptogenic cirrhosis (n = 49)	Virus-related cirrhosis (n = 105)	Autoimmune hepatitis (n = 76)	Total (n = 230)	
**Gender**	**Male, n (%)**	39 (79.6 %)	90 (85.7 %)	68 (89.5 %)	197 (85.7 %)	121 (86.4 %)
	**Female, n (%)**	10 (20.4 %)	15 (14.3 %)	8 (10.5 %)	33 (14.3 %)	19 (13.6 %)
**Age**	**Mean ± SD**	55.7 ± 9.6	61.3 ± 8.9	50.6 ± 10.5	57.3 ± 11.6	55.1 ± 10.3

**Fig. 1:**
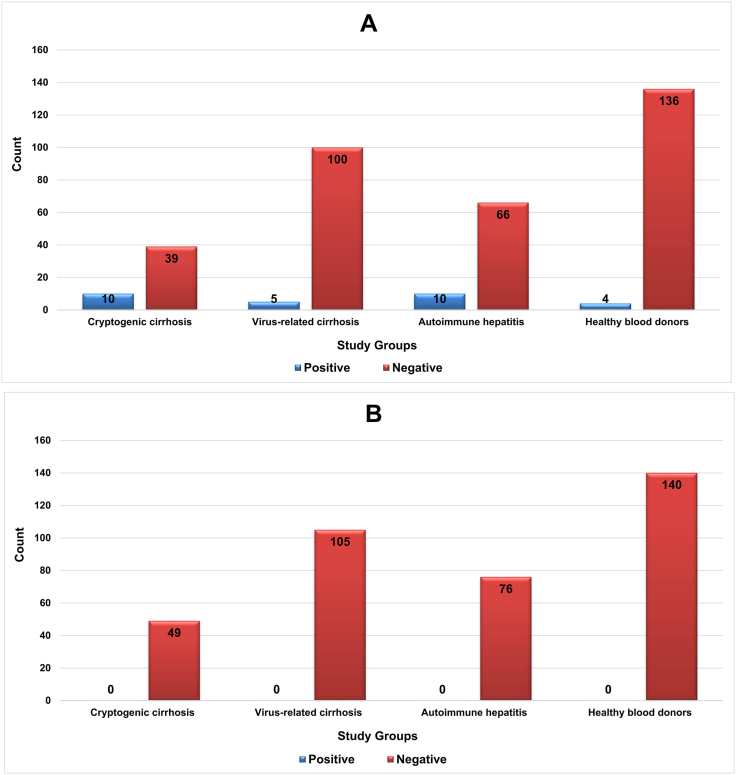
The *HHV-8* seropositivity rate in the study population. A) HHV-8 IgG antibody; B) HHV-8 genomic DNA.

**Fig. 2:**
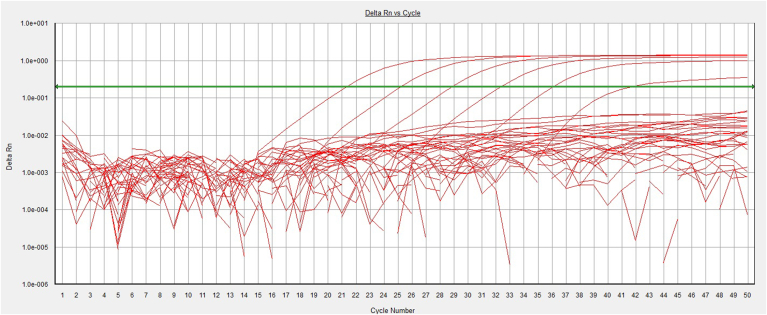
The amplification plot in the real-time PCR assay. The green line represents the threshold. The first to sixth peaks from the right correspond to the standards with concentrations of 2, 20, 200, 2,000, 20,000, and 200,000 copies/μL that were successfully amplified. However, samples from both the patient and control groups tested negative for the *HHV-8* genome. (For interpretation of the references to color in this figure legend, the reader is referred to the Web version of this article.)

Here, liver transplant candidates were classified into two groups: those with cryptogenic cirrhosis, those with virus-related cirrhosis, and those with autoimmune hepatitis. Cryptogenic cirrhosis is a term used for cirrhosis of unknown cause [7]. Virus-associated cirrhosis is a significant complication of liver disease. Numerous investigations have focused on the anticipation and control of hepatic decompensation among individuals suffering from cirrhosis induced by the hepatitis virus [31]. Autoimmune hepatitis is a persistent inflammatory disease of the liver characterized by elevated levels of aminotransferases, autoantibodies, hypergammaglobulinemia, and interface hepatitis. The determination of such disease is predicated on clinical and laboratory discoveries, encompassing heightened liver enzymes and hypergammaglobulinemia, the existence of autoantibodies, and corresponding histological abnormalities [32]. As shown in Fig. 1, HHV-8 IgG antibodies were detected in 25 patients (10.8 %) with pathologically proven cirrhosis and four healthy individuals (2.6 %). The results revealed a statistically significant difference between the two groups with respect to the seroprevalence of the *HHV-8* IgG antibody (p = 0.022). Moreover, the majority of the *HHV-8*-positive patients (10 out of 49, 20.4 %) had cryptogenic cirrhosis, followed by autoimmune hepatitis (10 out of 76, 13.1 %) and virus-related cirrhosis (5 out of 105, 4.7 %). The seropositivity of *HHV-8* was significantly different among the etiologies of liver cirrhosis (p = 0.011). Although the results revealed no significant difference between patients with virus-related cirrhosis and healthy individuals concerning the seroprevalence of *HHV-8* IgG antibodies (p = 0.503), patients with cryptogenic cirrhosis (p < 0.0001) and those with autoimmune hepatitis (p = 0.007) presented a greater rate of HHV-8 seropositivity than healthy controls. Despite the exquisite sensitivity of the real-time PCR assay, HHV-8 DNA was not detected in the sera of the cirrhotic patients or the control group (Fig. 2).

## 4 Discussion

Liver cirrhosis is a complex disease characterized by fibrosis that leads to impaired liver function and increased predisposition to HCC. The diagnostic criteria for cirrhosis have changed in the past few years because of improved noninvasive procedures, which help early diagnosis without liver biopsy. The diagnostic criteria for liver cirrhosis include clinical assessment along with imaging techniques and biopsy when needed. The abovementioned criteria have been further developed in recent years, and the focus has shifted to causes and complications [33,34].

Dead disease outbreaks and viral infections still pose important public health concerns in various societies because of great catastrophes in human history [[35], [36], [37], [38]]. Among different infectious agents, four nonhepatitis viruses, namely, *Epstein‒Barr virus* (*EBV*), human papilloma virus (*HPV*), *Merkel cell polyomavirus* (*MCPyV*), and *HHV-8*, have been recognized as oncogenes in solid organ transplants (SOTs). SOT recipients may be infected either before transplantation and reactivate the virus posttransplantation or acquire the virus as a donor-derived infection [39,40]. Hence, seroconversion of these viruses may account for disease manifestations in the transplantation setting, impact the outcome of SOT recipients, and cause great concern for transplant physicians [41].

*HHV-8* belongs to the human herpesvirus family and has both latent and lytic phases. Host immunity plays an important role in the control of *HHV-8* infection, as evidenced by the increased incidence of *HHV-8*-related diseases among immunosuppressed patients. Both nonneoplastic and neoplastic *HHV-8* disorders have been diagnosed in immunocompromised individuals. *HHV-8* can be transmitted at the time of organ donation and transplantation [40,42]. In a multicenter Italian study, the seroprevalence of *HHV-8* viremia was reported to be 4 % and 18 % in transplant donors and recipients, respectively [43]. The clinical importance of HHV-8 in transplant recipients is related not only to the transmission of viruses and/or reactivation of latent infection during immunosuppression but also to an increase in lethality. In other words, the intensive immunosuppression utilized to prevent graft rejection predisposes transplant recipients to chronic infections and the development of severe nonmalignant and malignant diseases [42,44]. For example, the risk of developing Kaposi's sarcoma, a rare tumor that occurs due to *HHV-8* infection, is 500–1000 times greater among organ transplant recipients than among the general population, depending on the type of transplanted organ. Posttransplant KS is a consequence of reactivation of latent infection in seropositive recipients or primary donor-derived infection in seronegative recipients. There are also some reports in the literature in which patients with donor-derived *HHV-8* infection died from the development of KS or associated complications. Therefore, preventive reduction of immunosuppression has been suggested in donor-positive/recipient-negative patients [20,40,42,43,45,46]. To date, most clinical studies on KS have been performed on kidney transplant recipients (possibly because of the larger number of kidney transplants). However, it may involve other organ transplant recipients, such as liver, heart, and lung recipients [47]. Generally, Kaposi sarcoma occurs less frequently after hematopoietic stem cell transplantation than after SOT [42]. Seroepidemiological studies have revealed an association between *HHV-8* infection and liver cirrhosis and its severity [17,48]. Nonetheless, there is a missing link between the prevalence of *HHV-8* infection and advanced liver disease. Therefore, the present research aimed to assess the frequency of *HHV-8* IgG antibody and DNA positivity among Iranian cirrhotic patients on the waiting list for liver transplantation, focusing on those related to cryptogenic cirrhosis, virus-related cirrhosis, and autoimmune hepatitis.

The findings revealed *HHV-8* IgG antibodies in 2.6 % of the healthy individuals, which is in line with the results of previous studies that revealed *HHV-8* antibodies in only 2 % of healthy Iranian blood donors [49]. Overall, epidemiological studies have demonstrated that *HHV-8* is not ubiquitous and that its prevalence varies considerably among different countries, ranging from low in most Asian countries (less than 3 %) [50] to low to moderate in Western countries (3–23 %) [51] and high in sub-Saharan Africa (up to 55 %) [52]. In addition to geographical factors, ethnicity, socioeconomic status, hygiene practices, and sexual behaviors might affect the prevalence of *HHV-8* viremia.

According to the results of the present study, the *HHV-8* IgG antibody could be detected in 10.8 % of the liver transplant candidates, which was significantly different from the rate observed in healthy individuals (2.6 %). Serologic screening for *HHV-8* viremia is not routinely included in pretransplant evaluation, even in endemic areas [42]. To date, the prevalence of *HHV-8* infection in cirrhotic patients has been determined in Taiwanese populations, with seropositivity ranging from 42 % to 49.5 % [17,48,53]. These rates are statistically higher than those reported in the present investigation. Taiwanese patients with hepatocellular carcinoma (HCC) have a greater incidence of *HHV-8* infection and higher anti-*HHV-8* antibody titers [18]. The high prevalence of *HHV-8* antibodies among the mentioned group could be explained by the vast prevalence of *HHV-8* in the general Taiwanese population, reaching 24 % [17,18,48]. A previous study that focused on pretransplant serological screening of *HHV-8* infection in SOT revealed that 33 % of the centers (17 centers) from a total of 51 transplant centers in 15 countries performed such serological screening [54].

In a study recently published on Iranian patients with cryptogenic cirrhosis, 11 out of 67 patients (16.4 %) were positive for *HHV-8* IgG antibodies, while *HHV-8* genomic DNA was detected in the plasma and peripheral blood mononuclear cell samples of 3 (4.5 %) and 5 (7.5 %) patients, respectively. In addition, 2.9 % of the healthy blood donors (3 out of 70) in this study were positive for *HHV-8* IgG antibodies, while viral DNA was not detected in the plasma or peripheral blood mononuclear cell samples [55]. The seroprevalence of *HHV-8* IgG antibodies in the present study was similar to the findings of the present study. The prevalence of *HHV-8* has been reported to be less than 3 % in the general population of Iran [49,56], which might, in part, explain the difference in the prevalence of HHV-8 between Taiwanese and Iranian cirrhotic patients. These findings highlight the public health significance of *HHV-8* and provide population-based epidemiological data that might improve the current understanding of *HHV-8* epidemiology in cirrhotic patients. Thus, it is reasonable to screen such patients for the possibility of *HHV-8* infection. DNA monitoring and serological screening are associated with the diagnosis of nonmalignant *HHV-8*-associated diseases [54].

Another interesting finding in the present study was the higher rate of *HHV-8* infection (20.4 %) in patients with cryptogenic cirrhosis, which represents a significant proportion of patients with end-stage liver disorders and is considered one of the most important indications for liver transplantation in the Iranian population [57]. The results also revealed a greater seroprevalence of *HHV-8* IgG antibodies in patients with autoimmune hepatitis than in healthy controls (13.1 % versus 2.6 %). However, the seroprevalence of *HHV-8* IgG antibodies was nearly significant in patients with virus-related cirrhosis and healthy individuals (4.7 % versus 2.6 %), which contrasts with the results obtained in Taiwanese patients [17,48,53]. In general, members of the *Herpesviridae* family, such as *HHV-8*, are involved in the development and progression of various autoimmune disorders through structural or functional mimicry and the development of latency in B lymphocytes [58]. The patients in the current study did not have a history of blood transfusion or high-risk issues that could lead to the transmission of blood-borne viruses. In addition, all the samples were negative for *HHV-8* genomic DNA, whereas some cirrhotic patients (n = 25) and healthy individuals (n = 4) were positive for *HHV-8* IgG. These results did not provide a sufficient basis regarding the high prevalence of *HHV-8* IgG antibodies as a real history of infection. Considering the low prevalence of *HHV-8* infection in the Iranian population [49,56], the high rate of *HHV-8* IgG antibody positivity in the cirrhotic patients in the present study, especially those with cryptogenic cirrhosis or autoimmune hepatitis, might support the hypothesis of antibody-mediated epitope mimicry with host proteins. In other words, patients may exhibit homology between *HHV-8* and autoantibody targets, which can be considered important clues in cross-reactive antibody production and can play a role in the high rate of seropositivity and possibly lead to liver injury. On the other hand, active replication of the viral genome generally results in lytic infection characterized by the release of new progeny virus particles. Given the high efficiency of the kit used for real-time PCR in this study, which can detect fewer than 100 copies of the target template, *HHV-8* should be detected in patient sera if the infection results from a primary infection. Even if HHV-8 is reactivated and latent viruses are switched to the lytic phase of replication, the frequency of *HHV-8* IgG antibody seropositivity does not change. Taking the abovementioned issues into account, neither hypotheses of primary infection nor reactivation of latent *HHV-8* infection are suggested; therefore, the antibody-mediated epitope mimicry hypothesis may have contributed to the high seroprevalence of *HHV-8* IgG antibody in the liver transplant candidates in the present study.

One of the cellular receptors hijacked by HHV-8 to gain access to cells is the EphA2 tyrosine kinase receptor. Briefly, the *HHV-8* envelope glycoprotein complex H and L (gH/gL) can bind with subnanomolar affinity to EphA2 *via* molecular mimicry of the cellular ligand of the receptor ephrin, which plays a pivotal role in the conserved gH residue E52 and the amino-terminal peptide of gL. In addition, the gH/gL complex functionally mimics the ephrin ligand by inducing the EphA2 receptor *via* its dimerization interface, thus triggering receptor signaling for cytoskeleton remodeling [9,59].

Considering the low to moderate prevalence of *HHV-8* infection in patients awaiting liver transplantation [60], targeted screening of liver transplant recipients and donors for the serological detection of *HHV-8* in combination with posttransplant viral load monitoring may be a useful prevention strategy. Since the present cirrhotic patients came from the most important liver transplant centers in Iran, the results can be generalized to the entire Iranian population, which can be of great importance for the prevention and control of disease manifestations. However, further intensive studies with larger sample sizes and longer follow-up periods are warranted to better understand the epidemiology of *HHV-8* viremia in liver transplant candidates with different etiological factors.

## 5 Conclusion

The high rate of *HHV-8* IgG antibody positivity in Iranian cirrhotic patients, especially those with cryptogenic cirrhosis or autoimmune hepatitis, combined with the undetectable viral load in these patients highlights the antibody-mediated epitope mimicry hypothesis involving host proteins, which might play a role in the development of advanced liver diseases. The results also highlighted the likelihood of previous *HHV-8* infection in patients with virus-related cirrhosis, which might be a challenge for physicians after liver transplantation. Since posttransplant reactivation of latent infection can be a life-threatening complication in transplant recipients, screening with anti-*HHV-8* antibodies and genomic DNA is appropriate for detecting latent and active infections and can reduce the rates of seroconversion and other complications associated with *HHV-8* infection, such as the development of neoplastic and nonneoplastic diseases. However, future studies with long-term follow-up are needed to assess the impact of real *HHV-8* infection and/or antibody-mediated epitope mimicry with host proteins on liver transplantation outcomes.

## Ethics and consent

The research protocol was approved by the local Ethics Committee of Shiraz University of Medical Sciences (approval nos. IR.SUMS.REC.1397.772 and IR.SUMS.MED.REC.1402.395). In addition, all the participants were required to provide written informed consent in accordance with the Declaration of Helsinki and its later amendments.

## Funding

This study was financially supported by grant No. 97-01-59-18186 and 28383 from the Vice-Chancellor for Research Affairs of Shiraz University of Medical Sciences, Shiraz, Iran.

## Data availability

The data that support the findings of this study are available from the corresponding author upon reasonable request.

## CRediT authorship contribution statement

**Javad Moayedi:** Writing – original draft, Software, Methodology, Formal analysis, Conceptualization. **Ava Hashempour:** Writing – review & editing, Supervision, Resources, Funding acquisition, Conceptualization. **Zahra Musavi:** Methodology, Investigation, Data curation. **Farzaneh Ghasabi:** Methodology, Investigation, Data curation. **Nastaran Khodadad:** Methodology, Investigation, Data curation. **Mohamad Ali Davarpanah:** Writing – review & editing, Resources, Funding acquisition, Conceptualization. **Ali Hasanshahi:** Methodology, Investigation, Data curation.

## Declaration of competing interest

The authors declared that they have no conflict of interest.
